# Occult Metastatic Melanoma Presenting as an Acute Coronary Syndrome

**DOI:** 10.21470/1678-9741-2017-0044

**Published:** 2017

**Authors:** Tiago R. Velho, Nádia Junqueira, André Sena, Hugo Ferreira, Catarina Carvalheiro, Nuno Guerra, Javier Gallego, Ângelo Nobre

**Affiliations:** 1 Cardiothoracic Surgery Department, Hospital de Santa Maria - CHLN, Lisboa, Portugal.

**Keywords:** Melanoma, Acute Coronary Syndrome, Minimally Invasive Surgical Procedures

## Abstract

Melanoma is a tumor that virtually involves any tissue and commonly metastasizes to the heart. It is usually not diagnosed because of the absent/nonspecific cardiac signs and symptoms. Herein, we present a case of a 41-year-old man without any cardiovascular risk factor, admitted to the emergency room with chest pain, diagnosed with a myocardial infarction. Due to the presence of a mass adjacent to the mitral valve on the cardiac ultrasound examination, causing mitral regurgitation, the patient was referred to surgery. Pathological analysis of the excised specimens diagnosed the melanoma. The chemotherapy was started and achieved a partial response. Cardiac metastases usually affect the myocardium, leaving the valves unaffected. In this case, the acute coronary syndrome was the first manifestation of the malignant melanoma. We highlight the high level of suspicion needed in these cases.

## INTRODUCTION

Melanoma is a tumor that arises from melanocytes or melanocyte precursors, and may virtually involve any tissue in the body^[1]^. It commonly metastasizes to the heart (40-45% of the cases)^[1]^, but usually cardiac involvement is only diagnosed post-mortem because of the absent or nonspecific cardiac signs and symptoms^[2]^. Secondary cardiac lesions are up to 100 times more frequent than primary tumors^[3]^. Although they usually tend to remain silent^[3]^, cardiac melanoma can cause serious mechanical (*e.g.* limitation of blood flow through the cardiac chambers) and electrical complications (usually associated with myocardial infiltrative masses) and embolization of the tumor^[4]^. In this article, we present a case of a patient admitted to the hospital with chest pain and diagnosis of myocardial infarction, with a ventricular mass identified. Histologic analysis after surgical resection revealed an occult metastatic melanoma.

## CASE REPORT

**Fig. 1:**
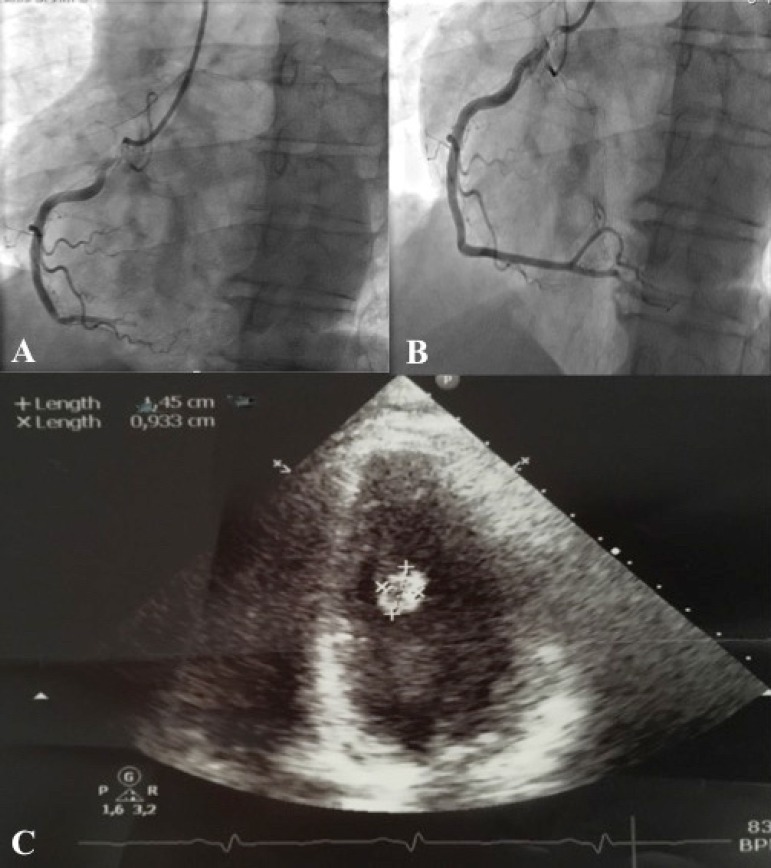
A) Coronariography showing right coronary occlusion. B) Final result after percutaneous treatment. C) Echocardiography showing a para-mitral valve mass of 1.5 cm.

A 41-year-old man without any cardiovascular risk factor presented to the emergency department with a 2-hour history of atypical chest pain. The patient had no history of previous episodes, or any other sign or symptom. At the examination, his pulse was arrhythmic at 125 beats/min. The electrocardiogram showed atrial fibrillation and 5 mm-ST-segment elevation on the inferior derivations. Troponin T was 3628 ng/L. The patient underwent immediate coronariography, and a thrombotic occlusion was visualized in the right coronary artery (Figure 1A), which was treated with aspiration, percutaneous balloon and abciximab (Figure 1B). The thrombus was not collected to histological analysis.

**Fig. 2:**
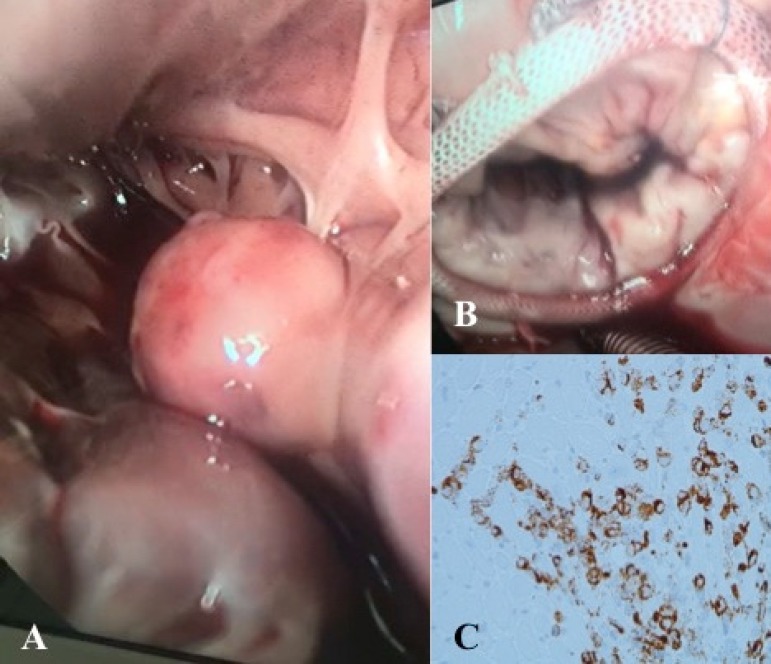
A) Para-mitral valve mass observed in the surgery. B) The mitral valve was surgically repaired with an annuloplasty. C) Histology of the myocardium with immunohistochemistry for the HMB-45 antigen.

The echocardiogram showed hypokinesia of the inferior and posterior walls with normal left ventricular function, and the presence of a 10 x 15 mm mass (assumed to be a thrombus) adjacent to the mitral valve, causing mild to moderate mitral regurgitation (Figure 1C). The mass was unaltered with anticoagulation for 48 hours, so the patient was proposed to surgery. The patient was transferred to the cardiothoracic surgery department and was submitted to resection of the ventricular tumor. An 8 cm mini-thoracotomy was performed on the right 5^th^ intercostal space and peripheral cannulation cardiopulmonary bypass was established. On the epicardial adipose tissue dark lesions were observed and excised for pathology. After left atriotomy, a large (2 x 2 cm), round and solid mass adjacent to the papillary muscle (Figure 2A) was causing mitral valve regurgitation. The mass was excised, including a partial resection of a papillary muscle, and sent for pathological examination. The mitral valve was surgically repaired with an annuloplasty (32 mm-ring) (Figure 2B). After annuloplasty, no regurgitation was observed during intraoperative echocardiogram.

Pathological examination revealed that undifferentiated tumor cells infiltrated both the papillary muscle and epicardial adipose tissue. The tumor cells were positive on the immunocytochemistry to melan-A, vimentin, MITF and HMB45 (negative to CKAE1/AE3, CK7, CK20, LCA, CD3, CD20, WT1 and calretinin), concluding that the tumor was a melanoma (Figure 2C). The presence of the BRAF-V600E mutation was detected.

The patient's postoperative course was uneventful. He was discharged on the fourth postoperative day and referred to the oncology department. The patient underwent a thoraco-abdominopelvic computed tomography, which revealed metastatic disease to the lungs, liver, skin and lymph nodes. The patient started chemotherapy with vemurafenib and cobimetinib, to which he remains with partial response.

## CONCLUSION

Although cardiac involvement in metastatic melanoma is frequent, symptomatic presentations are rare^[2]^. Most cases are not diagnosed *antemortem* due to the silent clinical course^[4]^.

Most metastasis are located in the myocardium and valvular structures are typically unaffected^[4]^. When searching for a primary focus of the metastatic melanoma, mucosal (including genital mucosal) and ophthalmologic examinations are essential and must not be disregarded.

Few articles have been published reporting cardiac metastasis of malignant melanoma with unknown primary origin. In this case, the acute coronary syndrome was its first manifestation, since the patient had no history of previous mucosal and/or skin lesions or surgery.

We highlight the essential high level of suspicion, since cardiac tumors should raise the possibility of being a metastatic melanoma.

**Table:** 

Authors' roles & responsibilities	
TRV	Substantial contributions to the conception or design of the work; drafting the work or revising it critically for important intellectual content; final approval of the version to be published
NJ	Substantial contributions to the conception or design of the work; drafting the work or revising it critically for important intellectual content; final approval of the version to be published
AS	Substantial contributions to the conception or design of the work; drafting the work or revising it critically for important intellectual content; final approval of the version to be published
HF	Substantial contributions to the conception or design of the work; drafting the work or revising it critically for important intellectual content; final approval of the version to be published
CC	Substantial contributions to the conception or design of the work; drafting the work or revising it critically for important intellectual content; final approval of the version to be published
NG	Substantial contributions to the conception or design of the work; drafting the work or revising it critically for important intellectual content; final approval of the version to be published
JG	Substantial contributions to the conception or design of the work; drafting the work or revising it critically for important intellectual content; final approval of the version to be published
AN	Substantial contributions to the conception or design of the work; drafting the work or revising it critically for important intellectual content; final approval of the version to be published
